# Using photoelectron spectroscopy to measure resonant inelastic X-ray scattering: a computational investigation

**DOI:** 10.1107/S1600577521011917

**Published:** 2022-01-01

**Authors:** Daniel J. Higley, Hirohito Ogasawara, Sioan Zohar, Georgi L. Dakovski

**Affiliations:** a SLAC National Accelerator Laboratory, 2575 Sand Hill Road, Menlo Park, CA 94025, USA

**Keywords:** resonant inelastic X-ray scattering, photoelectron spectroscopy, deconvolution, data analysis, X-ray spectroscopy

## Abstract

Resonant inelastic X-ray scattering (RIXS) has become an important scientific tool. Nonetheless, conventional high-resolution (few hundred meV or less) RIXS measurements, especially in the soft X-ray range, require low-throughput grating spectrometers, which limits measurement accuracy. Here, the performance of a different method for measuring RIXS, *i.e.* photoelectron spectrometry for analysis of X-rays (PAX), is computationally investigated. This method transforms the X-ray measurement problem of RIXS to an electron measurement problem, enabling use of high-throughput, compact electron spectrometers. X-rays to be measured are incident on a converter material and the energy distribution of the resultant photoelectrons, the PAX spectrum, is measured with an electron spectrometer. A deconvolution algorithm for analysis of such PAX data is proposed. It is shown that the deconvolution algorithm works well on data recorded with ∼0.5 eV resolution. Additional simulations show the potential of PAX for estimation of RIXS features with smaller widths. For simulations using the 3*d* levels of Ag as a converter material, and with 10^5^ simulated detected electrons, it is estimated that features with a few hundred meV width can be accurately estimated in a model RIXS spectrum. For simulations using a sharp Fermi edge to encode RIXS spectra, it is estimated that one can accurately distinguish 100 meV FWHM peaks separated by 45 meV with 10^5^ simulated detected electrons that were photoemitted from within 0.4 eV of the Fermi level.

## Abstract 2

Using a deconvolution algorithm, it is shown that resonant inelastic X-ray scattering (RIXS) spectra can be reliably estimated from measurements of photoelectrons created through absorption of RIXS photons in a chosen material. The method works on previously reported data with ∼0.5 eV resolution, and simulations show its potential for accurately estimating spectral features with hundreds of meV or smaller width.

## 1. Introduction

Resonant inelastic X-ray scattering (RIXS) has emerged as a powerful technique to study elementary excitations (Ament *et al.*, 2011 ▸). RIXS probes excitations via core–valence transitions with element-specific energies, which allows one to tune the elemental locations being probed through the incident X-ray photon energy. Because of the large momentum of X-rays, RIXS is able to probe the dispersion of elementary excitations in solids, unlike lower-energy optical photons. Further, in contrast to other X-ray-based spectroscopies, the energy resolution of RIXS is not limited by short core hole lifetimes. Because of these strengths, much effort has been devoted to developing RIXS capabilities. In the soft X-ray range, X-ray grating and synchrotron light source development has enabled RIXS measurements with energy resolution better than 100 meV (Brookes *et al.*, 2018 ▸; Dvorak *et al.*, 2016 ▸). Having these high energy resolutions is particularly important for studies of solids where characteristic energies of many important excitations are around 100 meV or less (Ament *et al.*, 2011 ▸; Chaix *et al.*, 2017 ▸). Leveraging these capabilities, RIXS studies have given new insights in wide-ranging topics including solid state physics (Ament *et al.*, 2011 ▸; Le Tacon *et al.*, 2011 ▸; Schlappa *et al.*, 2012 ▸; Chaix *et al.*, 2017 ▸), nanoparticles (Liu *et al.*, 2017 ▸), interfaces (Rajasekaran *et al.*, 2012 ▸), batteries (House *et al.*, 2020 ▸; Firouzi *et al.*, 2018 ▸), liquids (Wernet *et al.*, 2015 ▸) and gases (Hennies *et al.*, 2010 ▸).

Even more information could be gleaned from RIXS if one could make faster and more accurate measurements that maintained hundreds of meV or better resolution. For example, high-resolution time-resolved RIXS measurements probe dynamics of elementary excitations and can discriminate well between different states that occur in sample evolution (Wernet *et al.*, 2015 ▸), but require recording many accurate spectra. Unfortunately, grating spectrometers detect only one X-ray photon for every ∼10^7^ or more scattered RIXS photons (Qiao *et al.*, 2017 ▸; Ghiringhelli & Braicovich, 2013 ▸; Dakovski *et al.*, 2017 ▸), making such studies very challenging. Work has been done towards improving this. Transition edge sensors (Uhlig *et al.*, 2015 ▸) and off-axis zone plates (Marschall *et al.*, 2017 ▸) can make more accurate RIXS measurements than traditional instrumentation in certain cases, but so-far demonstrated resolutions are significantly more than 0.5 eV. More information could be gleaned from RIXS if one could make faster and more accurate measurements that maintained hundreds of meV or better resolution. Here, we investigate an alternative technique that leverages electron spectroscopy for RIXS.

Coinciding with the rise and development of RIXS, the capabilities of electron spectrometers have also greatly increased (Damascelli *et al.*, 2003 ▸). Photoelectron spectrometers can now have energy resolutions better than 20 meV for 500 eV kinetic energy electrons (Seidel *et al.*, 2017 ▸). The collection efficiency, and thus achievable signal-to-noise ratio with a given number of particles emitted from a sample, can be much higher for electron spectrometers than X-ray spectrometers with comparable resolutions. Further, electron spectrometers are not as sensitive to the vibrations and movements that can impact the performance of large X-ray spectrometers. These features are largely due to the ease with which electrons can be manipulated, owing to their charge, in comparison with neutral X-rays. Thus if one can transform the X-ray measurement problem of RIXS to an electron measurement problem, then there could be gains in count rates, and instrumentation simplicity.

**Figure 1:**
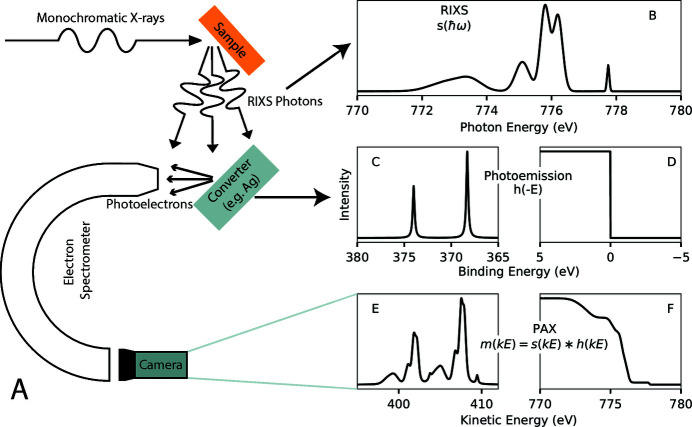
Concept of PAX for estimating RIXS spectra. (A) Experimental schematic. Monochromatic X-rays are incident on a sample which emits RIXS photons. These photons are directed onto a converter material resulting in photoemission. The energy distribution of these photoelectrons is detected with an electron spectrometer giving a PAX spectrum. We estimate the RIXS spectrum from the measured PAX spectrum and knowledge of the converter material photoemission spectrum. (B) Example model ground truth RIXS spectrum at the Co *L*_3_-edge (778 eV incident photon energy). (C) Ag 3*d*-like model photoemission spectrum. (D) Sharp Fermi edge model photoemission spectrum, such as for Au. (E) Resultant PAX spectrum without noise for the X-ray spectrum in (B) and the photoemission spectrum in (C). (F) Resultant PAX spectrum without noise for the X-ray spectrum in (B) and the photoemission spectrum in (D).

Recently, Dakovski *et al.* (2017 ▸) proposed doing exactly that to measure RIXS with hundreds of meV or better resolution through photoelectron spectrometry for analysis of X-rays (PAX) (Krause, 1965 ▸; Ebel, 1975 ▸). Fig. 1 ▸ gives an overview of this technique, which makes use of sharp photoemission features that occur in the photoemission spectra of materials such as Ag, Au, Pt or Al when measured with monochromatic incident X-ray radiation. Fig. 1 ▸(A) shows an experimental schematic. X-rays to be measured [Fig. 1 ▸(B)] are incident on a converter system, where absorption of the X-rays generates photoelectrons. The converter material is assumed to give some photoemission spectrum when measured with monochromatic X-ray radiation, *xps*(*BE*), as a function of binding energy, *BE* [Fig. 1 ▸(C) shows an example Ag 3*d* photoemission spectrum, while Fig. 1 ▸(D) shows an example sharp Fermi edge]. The emitted photoelectrons are then detected with a photoelectron spectrometer. We call the resultant electron spectrum the PAX spectrum [Figs. 1 ▸(E) or 1(F)]. The expected shape of the PAX spectrum is given by a convolution with *h*(*E*) = *xps*(−*E*) acting as an impulse response function. Convolving the spectrum of X-rays incident on the converter material [Fig. 1 ▸(B)] with *h*(*E*) [Figs. 1 ▸(C) or 1(D)] approximately gives the expected value of the PAX spectrum [Fig. 1 ▸(E) or 1(F)], 

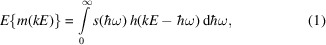

where we only integrate over physically realistic positive photon energies. Here, *kE* is the electron kinetic energy, *s*(ℏω) is the X-ray spectrum incident on the converter material as a function of photon energy, ℏω, and *m*(*kE*) is a measured PAX spectrum.

Given a PAX spectrum, the ground truth X-ray spectrum, *s*(ℏω), can be estimated directly through deconvolution or in a parameterized form such as a sum of peaks. (The ground truth X-ray spectrum is the X-ray spectrum that would be measured without noise and with perfect resolution.) The decomposition of RIXS spectra into a sum of peaks is a natural method already widely used for traditionally recorded RIXS spectra as the parameters of these peaks can be directly linked to physical characteristics of the matter under study (Ament *et al.*, 2011 ▸). For more complex RIXS spectra, or cases where less is known about the form of a RIXS spectrum before measurement, the more general case of deconvolution may be more appropriate or would be a first step in further decomposition of a RIXS spectrum into elementary features.

While Dakovski *et al.* (2017 ▸) demonstrated the possibility of recording PAX spectra for RIXS with moderate resolution and estimating the corresponding RIXS spectra as a sum of peaks, further development is required for PAX to be a proven high-resolution RIXS technique. First, a general algorithm for faithfully reconstructing X-ray spectra from PAX measurements is needed. The algorithm proposed by Dakovski *et al.* (2017 ▸) works when spectral line shapes and their photon energy distribution are priorly known in order to estimate the spectra as a sum of Gaussian functions. Without this information, spectral features will be poorly deconvoluted. Second, a quantitative assessment of the potential of PAX for measurement of X-ray spectra would show where PAX could be most useful. Finally, an experimental demonstration of the ability of PAX to perform a high-resolution RIXS study is needed. Here, we fill the first two of these gaps by proposing an algorithm for analyzing PAX data, then showing the capability of this algorithm on the data from Dakovski *et al.* (2017 ▸) and finally showing the potential of PAX for estimating finer RIXS features through simulations. The algorithm we propose is a regularized maximum-likelihood estimation algorithm where the optimal regularization strength is estimated in a self-supervised manner from PAX data (summarized later in Algorithms 1 and 2).

The rest of this report is organized as follows. In Section 2, we discuss the considerations for choosing a converter material for PAX, and explain why the Ag 3*d* lines and a sharp Fermi edge are compelling cases. In Section 3, we describe the deconvolution algorithm we propose for estimating RIXS spectra from PAX data. In Section 4 we show the performance of this algorithm on the experimental data of Dakovski *et al.* (2017 ▸) as well as the simulated performance of PAX in estimating hundreds of meV or finer model RIXS features. The results on the data from Dakovski *et al.* (2017 ▸) demonstrate the capability of PAX to estimate RIXS structure on the ∼eV scale with a general deconvolution algorithm. Further, we find that, in simulations under reasonable experimental conditions using the Ag 3*d* levels as a photoemission converter, PAX can accurately estimate the width of few hundred meV features when 10^5^ electrons are simulated to be detected in the measured PAX spectrum. Under more challenging to realize conditions, simulations using a sharp Fermi edge photoemission converter show potential for PAX in estimating finer features. Finally, in Section 5 we conclude and give an outlook for future investigations.

## 2. Choice of converter material

The converter material plays a key role in PAX measurements. Converter materials with high conversion efficiency and narrow photoemission lines are desirable. Higher conversion efficiencies give higher numbers of detected electrons and, thus, higher signal-to-noise ratios. Narrow photoemission lines enable retrieval of narrow X-ray spectral features with a reasonable number of detected electrons. For thick converter materials with near normal incidence of X-rays and emission of photoelectrons, the conversion efficiency of X-rays to photoelectrons can be approximated as (Henke, 1972 ▸) 

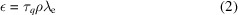

where τ_*q*_ is the effective ionization cross-section for the creation of photoelectrons from subshell *q*, ρ is the number per unit volume of atoms within the sample which can emit photoelectrons from the subshell used for PAX, and λ_e_ is the electron mean free path at the kinetic energy of the relevant photoelectrons.

**Figure 2:**
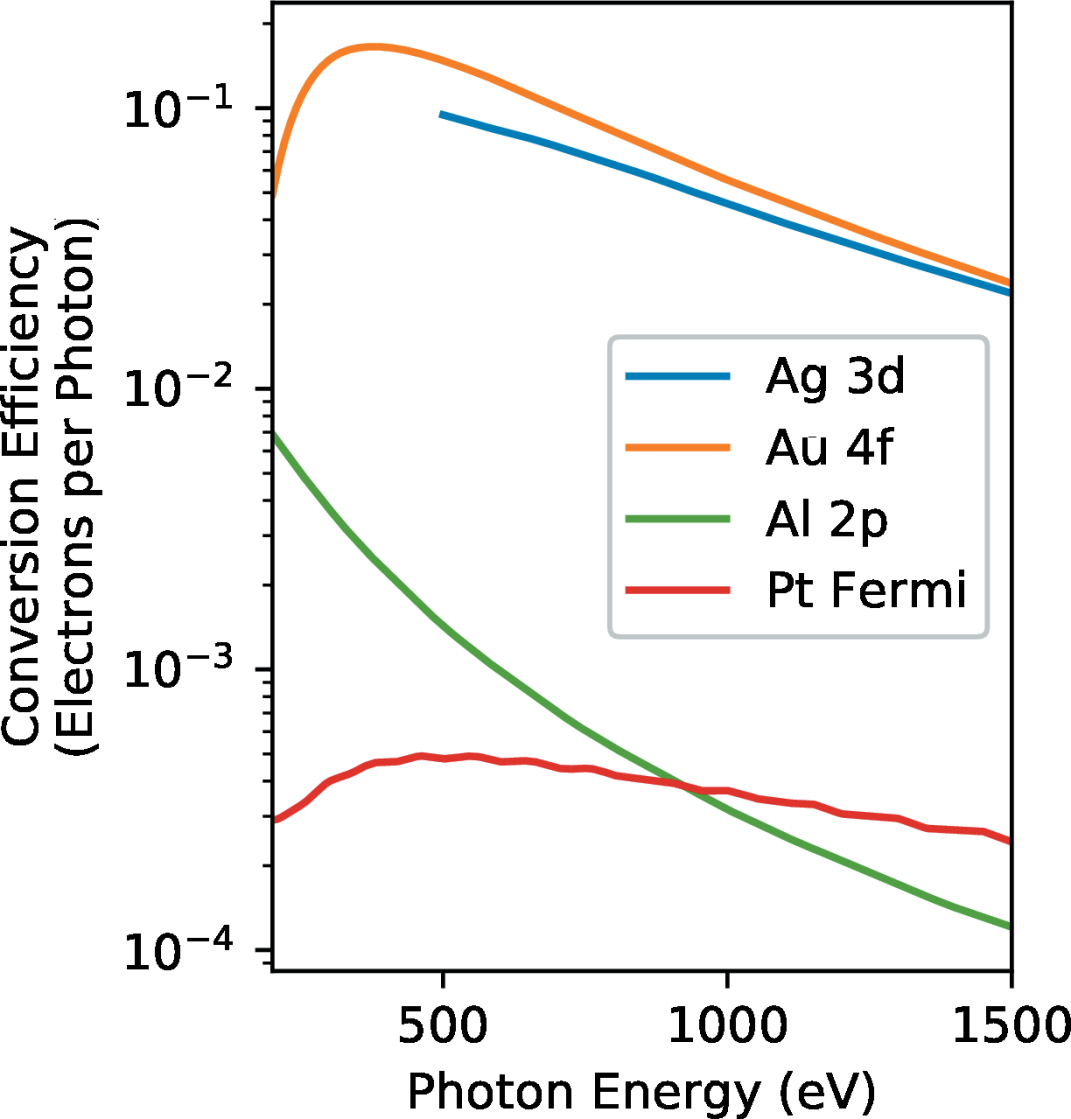
Photoemission quantum efficiency of some electronic subshells with potential for PAX.

In Fig. 2 ▸ we show the conversion efficiency estimated with equation (2) for some promising cases. For these calculations subshell photoemission cross sections were taken from Yeh & Lindau (1985 ▸), electron mean free paths for Ag and Au from Tanuma *et al.* (2002 ▸), electron mean free paths for Al and Pt from Shinotsuka *et al.* (2015 ▸), and atoms per unit volume calculated from values from Rumble (2019 ▸). From Fig. 2 ▸, we see that, in the soft X-ray range, conversion efficiencies of nearly 10% with photoemission linewidths of a few hundred meV are possible using the Au 4*f* or Ag 3*d* lines. Narrower photoemission features are available at the expense of a reduced conversion efficiency (such as Al 2*p* and Pt Fermi level photoemission shown in Fig. 2 ▸ and Table 1 ▸.

Photoemission feature widths are intrinsic properties of potential PAX converter materials. With perfectly accurate measurements, the width of features can be deconvoluted independently from the photoemission feature width. In practice, a measurement detects only a finite number of electrons. Therefore, the narrowest of photoemission feature widths set fundamental limits on the widths of spectral features that can be robustly estimated through PAX for a given number of detected electrons. For core level photoemission features, the natural width of the level gives a lower limit. This reaches the small value of 10 meV for the *L*-shell of Na (Riffe *et al.*, 1991 ▸). Widths are broadened from that fundamental limit in real matter though. The Al 2*p* levels have very small measured core level widths of ∼60 meV FWHM (Borg *et al.*, 2004 ▸). Thus, the fundamental limit of PAX resolution using core level photoemission is a maximum of 60 meV and minimum of 10 meV. Valence levels can have even smaller widths. The Xe 5*p*_3/2_ levels are widely used for characterizing the resolution of electron instrumentation due to their having a measured width as small as 3.4 meV (Mårtensson *et al.*, 1994 ▸). Thus, the maximum fundamental limit of PAX resolution using valence photoemission is 3.4 meV. This, however, comes at the expense of lower conversion efficiency than for core levels.

## 3. An algorithm for deconvolving PAX spectra

In principle, a similar convolution equation to equation (1) describes the measured signal in many X-ray spectroscopies. The signal of interest, such as a RIXS spectrum or X-ray absorption spectrum, is convolved with an instrument response function and an intrinsic broadening function to give the measured spectrum. Thus, more accurate X-ray spectra can often be retrieved through deconvolution of measured results (Ebel & Gurker, 1975 ▸; Fister *et al.*, 2007 ▸; Laverock *et al.*, 2011 ▸). It is not typical to analyze such spectra using deconvolution, however. This is because these convolutions only broaden the measured spectra, and measured spectra are still interpretable as a simple blurring of the true spectrum. For PAX, however, the measured spectrum is typically convolved with a more complicated function than a single peak. The converter material photoelectron spectrum could consist of, for example, two narrow peaks and a non-uniform background, as is common for core levels. It may not be easy to infer the original X-ray spectrum from the measured PAX spectrum in these cases. Thus, while deconvolution is an optional step in traditional X-ray spectroscopies, it is important for PAX measurements.

### 3.1. Model of PAX spectra

Equation (1) gives the expected value of a PAX measurement in the case that the PAX spectrum is measured at every electron kinetic energy. In reality, the measured PAX spectrum, *m*[*kE*] is discrete with each measured point integrating over a range of electron kinetic energies. Thus, the expected value of the measured PAX spectrum is approximately given by a discrete convolution, 

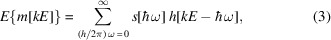

where *s*[ℏω] and *h*[−*BE*] are the discretized versions of the X-ray spectrum incident on the converter material and the photoemission spectrum of the converter material.

Equation (3) extends over an infinite range, but, fortunately, experimental circumstances can be chosen so that the summation is non-negligible only over a finite and experimentally tractable range. We assume that we want to estimate a RIXS spectrum from ℏω_min_ to ℏω_max_ and that we want to use photoemission features extending from at least *BE*_min_ to *BE*_max_ in the measurement. These ranges give a PAX spectrum extending from *kE*_min_ = ℏω_min_ − *BE*_max_ through *kE*_max_ = ℏω_max_ − *BE*_min_. In order to accurately model this PAX spectrum we must keep all X-ray photon energies in equation (3) that contribute non-negligibly to the PAX spectrum over this range. X-rays with energies higher than some cutoff ℏω_+_ give negligible contributions (a typical cutoff may be a few hundred meV above the incident X-ray energy). The lower limit of photon energies that contribute to the PAX spectrum is set through the lowest binding energy that contributes significantly to the PAX spectrum, *BE*_−_. For example, if one is using valence photoemission features, this limit on binding energies is set by the typical restriction of significant photoemission intensity to positive binding energies. This sets the lower limit ℏω_−_ = *kE*_min_ + *BE*_−_ on the photon energies of X-rays that will contribute to the PAX spectrum.

With these range limitations, equation (3) is simplified to 

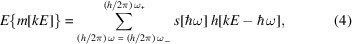

which is practical to analyze for PAX. It is convenient to write this as 

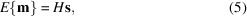

where **m** is a column vector whose entries are the measured PAX spectrum, **s** is another column vector whose entries are the desired X-ray spectrum, and *H* is a Toeplitz matrix such that the entries of *H* **s** are the same as the discrete convolution *h* * *s*.

We assume we are in a regime where shot noise is the dominant noise. In this case, the measured PAX spectrum can be approximated by a Poisson process. The probability of measuring *b* counts for a kinetic energy bin with expected value *a* is 

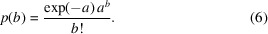

The probability of measuring a PAX spectrum **m** is then 

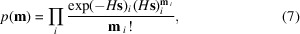

where **x**_*i*_ denotes the *i*th element of **x**.

We note that a matrix equation like equation (5) holds as a description of the expected value of a PAX spectrum even when the photoemission spectrum of the converter material is dependent on the incident photon energy. Thus, the methods we describe below can still be used to estimate a RIXS spectrum in such cases, albeit with likely less computational efficiency.

### 3.2. Regularized maximum-likelihood estimation for estimating RIXS with PAX

We now describe how we estimate a ground truth RIXS spectrum given a PAX data set. We assume that we have a set of PAX spectra recorded under statistically identical conditions as well as a high-accuracy measurement of the photoemission spectrum of the converter material recorded with monochromatic incident X-ray radiation. Neglecting noise of the photoemission spectrum is an acceptable approximation because the photoemission spectrum can be measured with direct photoemission which has orders of magnitude higher count rate than a PAX measurement. We estimate the ground truth RIXS spectrum from these data using regularized maximum-likelihood estimation. The maximum-likelihood estimate of the ground truth RIXS spectrum is the spectrum which maximizes the probability of measuring the actually measured PAX spectrum. Regularization prevents the estimate from having finer structure than is warranted for the quality of the data.

For the probability given in equation (7), the negative log-likelihood of **s** is 

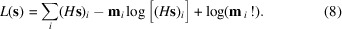

The gradient of this with respect to **s** is 

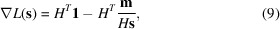

where **1** is a vector where all the entries are 1 and with dimension such that the equation it appears in is valid, and *x* ^*T*^ denotes the transpose of *x*. Having this gradient, we can iteratively minimize the negative log-likelihood (maximizing the likelihood) with the scaled gradient iteration (Bertero *et al.*, 2009 ▸) 

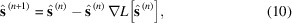

where 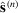 is the estimate of **s** after *n* iterations. This gives the iteration 

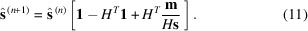

This is equivalent to 

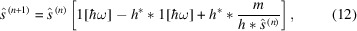

where *h* ^*^ is the photoemission impulse response function with the order of the entries reversed and all the entries of 1[ℏω] are 1. This iteration requires a starting point, 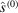 . For this, we used a smoothed version of the measured PAX spectrum.

If *h* ^*^ * 1[ℏω] = 1[ℏω], then equation (12) simplifies to the well known Lucy–Richardson algorithm (Richardson, 1972 ▸; Lucy, 1974 ▸; Shepp & Vardi, 1982 ▸). The Lucy–Richardson algorithm and its variants have been widely used in imaging (Bertero *et al.*, 2009 ▸; Dey *et al.*, 2006 ▸; Starck *et al.*, 2002 ▸) and spectroscopy (Fister *et al.*, 2007 ▸). For PAX, however, we often will not be able to reduce equation (12) to the Lucy–Richardson algorithm as photoemission spectra used for PAX can be non-negligible over a wide range.

It is a well known problem that algorithms like equation (12) amplify high-frequency noise when they are used without regularization (White, 1994 ▸; Bertero *et al.*, 2009 ▸). This is essentially a result of the reduction in strength of high-frequency components relative to low-frequency components after convolution with an extended function. Accurately inferring the pre-convolution strength of these high-frequency components requires a more accurate measure of their strength in the post-convolution data then their lower-frequency counterparts.

Various regularization schemes have been proposed to avoid the amplification of high-frequency noise encountered in such algorithms. This is typically achieved by enforcing some degree of smoothness of the deconvolved result. Regularization by stopping iterations after certain criteria have been met (Reeves, 1995 ▸), damping the effect of iterations that do not improve the reconstruction (White, 1994 ▸), and total variation regularization (Dey *et al.*, 2006 ▸) has been proposed. A method of regularization well suited to our case was proposed by Fister *et al.* (2007 ▸) for application to Lucy–Richardson deconvolution of X-ray absorption and inelastic X-ray scattering spectra. In this algorithm, the iterative deconvolution is stabilized against high-frequency noise amplification by convolution with a Gaussian function after each iteration. Applying this to our algorithm gives a regularized version of equation (12), 

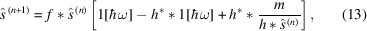

where *f*(*x*) is a Gaussian function with unit integrated amplitude, 

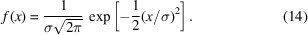

The width of the Gaussian, σ, constrains the maximum roughness of deconvolved spectra and thus sets the regularization strength (it acts as a hyperparameter in the deconvolution algorithm). Smaller regularization strengths allow for more roughness in the deconvolved spectra than larger regularization strengths.

**Figure 3:**
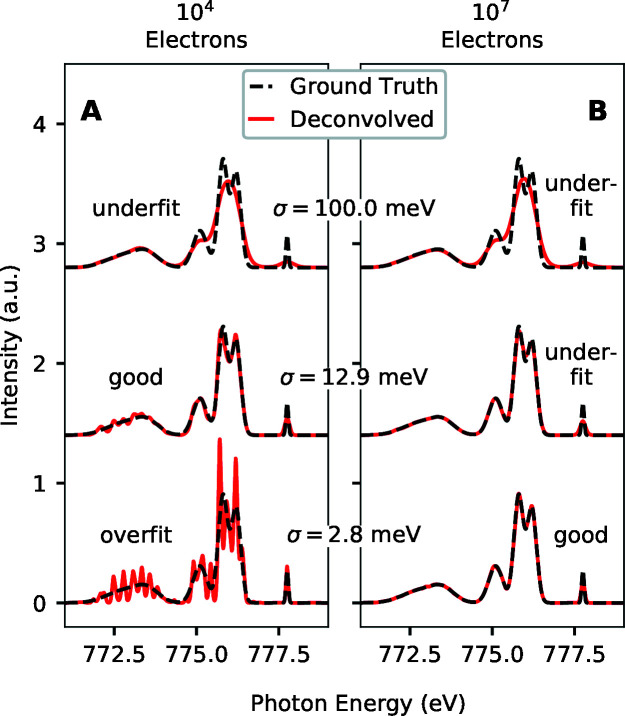
Effect of regularization strength on deconvolved PAX spectra. (Spectra with different regularization strengths are vertically offset for clarity.) (A) Ground truth and deconvolved spectra for different regularization strengths and 10^4^ simulated detected electrons. (B) Same as part A, except for 10^7^ simulated detected electrons.

Fig. 3 ▸ shows the effect of regularization strength on the deconvolved spectra for simulations using the model ground truth spectrum shown in Fig. 1 ▸(*a*), and model Ag 3*d* photoemission spectrum shown in Fig. 1 ▸(*b*). We used an energy separation of 10 meV between points for all simulations using the Ag 3*d* levels as a photoemission converter. Part A of Fig. 3 ▸ shows results for 10^4^ simulated detected electrons, while part B shows analogous results for 10^7^ simulated detected electrons. In each case, deconvolved and ground truth spectra are shown with regularization strength decreasing from top to bottom. As the regularization strength decreases, the deconvolved spectra attain more detail and increasingly finer structure is seen. This comes at the expense, however, of more statistical variation in the deconvolved spectra. For the case with 10^4^ simulated detected electrons, the deconvolved spectra accurately estimate increasingly fine spectral features with smaller regularization strengths except for the smallest regularization strength of σ = 2.8 meV. For that case, the deconvolved spectrum has fine features, but they do not accurately reflect the ground truth spectrum on this scale. In contrast, with 10^7^ simulated detected electrons, as shown in Fig. 3 ▸(B), the deconvolved spectra accurately reflect the ground truth spectrum smoothed to an extent given by the particular regularization strength even for the smallest regularization strength shown. Thus, the optimal regularization strength is smaller for 10^7^ simulated detected electrons than for 10^4^ simulated detected electrons. More generally, the best regularization strength decreases with increasing numbers of simulated detected electrons, but also depends on the converter material photoemission spectrum and the ground truth X-ray spectrum.

### 3.3. Estimating the optimal regularization strength

We now show how we can estimate the optimal regularization strength. Fister *et al.* (2007 ▸) proposed one method of choosing the regularization based on the smallest expected feature in the ground truth spectrum. Since we do not always know this before measurements, we use a more general procedure here. We define the optimal regularization strength as that which minimizes the root mean squared error (RMSE) of a deconvolved spectrum with respect to the ground truth it estimates, 

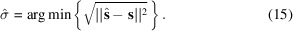

**Figure 4:**
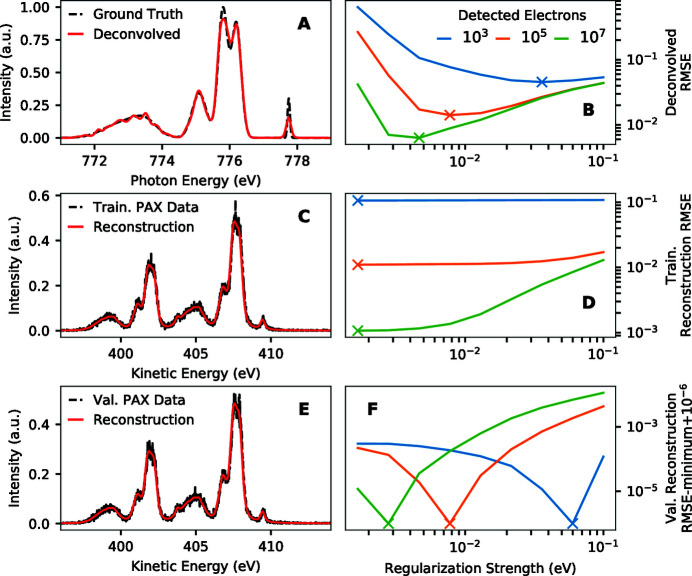
Illustration of how the quality of reconstruction of PAX spectra from deconvolved spectra can be used to estimate the optimal regularization strength. The panels on the left show example simulated data with 10^5^ simulated detected electrons and a regularization strength of 7.7 meV. (A) Example ground truth spectrum and an estimate of it obtained by deconvolving simulated data. (B) Corresponding root mean squared error (RMSE) of the deconvolved spectrum as a function of the regularization strength and the number of simulated detected electrons. (C) PAX spectrum obtained by averaging a training set of data (data that were used in deconvolution) and its reconstruction from the deconvolved result. The incident photon energy of 778 eV (Co *L*_3_) combined with the Ag 3*d* binding energies near 370 eV (Panaccione *et al.*, 2005 ▸) give electron kinetic energies near 405 eV in the PAX spectrum. (D) Corresponding RMSE of the reconstruction of the training data for the same parameters as (A). (E) PAX spectrum obtained by averaging a validation set of data (data that were not used in deconvolution) and its reconstruction from the deconvolved result. (F) Corresponding RMSE of the validation data for the same parameters as (A). The data of panel (F) are shown with the minimum of each curve subtracted and a small offset added in order to highlight the locations of minima. Since the validation reconstruction error is minimized at similar regularization strengths as the deconvolved error, we can estimate the optimal regularization strength from the validation reconstruction error.

Fig. 4 ▸ shows the dependence of different statistics on the regularization strength and number of simulated detected electrons. These simulations were carried out using the Ag 3*d* photoemission spectrum shown in Fig. 1 ▸(C) as a model photoemission converter and the model RIXS spectrum of Fig. 1 ▸(A) as a ground truth spectrum to estimate. The results shown in Fig. 4 ▸ can be used to assess the potential of the different statistics in estimating the optimal regularization strength.

Fig. 4 ▸(A) shows an example ground truth spectrum and a deconvolved estimate of it from simulated PAX data. Calculating the RMSE of such deconvolved spectra as a function of the regularization strength and number of simulated detected electrons results in the curves shown in Fig. 4 ▸(B). This deconvolved RMSE decreases with increasing regularization strength down to the minimum at the optimal regularization strength, where it then gradually increases with increasing regularization strength. The optimal regularization strength is smaller for higher numbers of simulated detected electrons. Unfortunately, this deconvolved RMSE is not experimentally accessible as the ground truth spectrum is generally unknown.

Instead of trying to determine the optimal regularization strength through direct assessment of deconvolved error, we can assess how well our model reconstructs PAX spectra from deconvolved spectra as a proxy for the accuracy of deconvolved spectra. In other words, we can compare the convolution of a deconvolved spectrum with the corresponding photoemission impulse response function with recorded PAX data. If the deconvolution is perfect and there is no noise, these spectra should be the same. It has been shown in previous deconvolution studies that such a comparison can allow one to estimate the optimal regularization strength (Reeves, 1992 ▸; Wahba & Wang, 1990 ▸). In making these assessments, we distinguish between two cases. In the first case, we compare a reconstruction of PAX data with a training PAX spectrum (the same spectrum that was used as input for deconvolution). In the second case, we compare a reconstruction of PAX data with a validation PAX spectrum (a spectrum simulated with statistically identical conditions as the training spectrum, but that was not used as part of that deconvolution).

Fig. 4 ▸(C) shows a comparison of a reconstruction of a PAX spectrum with a training PAX spectrum. Fig. 4 ▸(D) shows the RMSE of such reconstructions as a function of regularization strength and number of detected electrons. Unfortunately, as seen there, this statistic only decreases with decreasing regularization strength and is not minimized at the same locations as the deconvolved RMSE. Thus we cannot use the minimum of this statistic to estimate the optimal regularization parameter. Deconvolutions with too small regularization strengths closely fit fine features of the recorded PAX spectrum even though fitting so closely is not warranted given the noise in the data. This is an example of overfitting. To avoid this problem, we can assess the performance of the deconvolution on data that were not used as input to the deconvolution (James *et al.*, 2013 ▸).

Fig. 4 ▸(E) compares the PAX reconstruction with a validation PAX spectrum. Fig. 4 ▸(F) shows the dependence of the RMSE of the reconstruction with respect to the validation PAX spectrum on the regularization strength and the number of simulated detected electrons. In each case, this statistic is minimized near the optimal regularization strengths. Unlike the RMSE of deconvolved data shown in Fig. 4 ▸(B), this statistic is experimentally accessible, and thus we use it to estimate the optimal regularization strength.

### 3.4. Stopping criterion

To perform iterative deconvolution, it is necessary to decide when a sufficient number of iterations have been completed. To choose such a stopping criterion, we recognized that (1) we wanted to perform sufficient iterations for the deconvolution with the optimal regularization strength to converge to near its asymptotic values, and (2) we wanted the number of iterations to be high enough for the optimal regularization strength to be closely approximated by that which minimizes the validation reconstruction RMSE.

**Figure 5:**
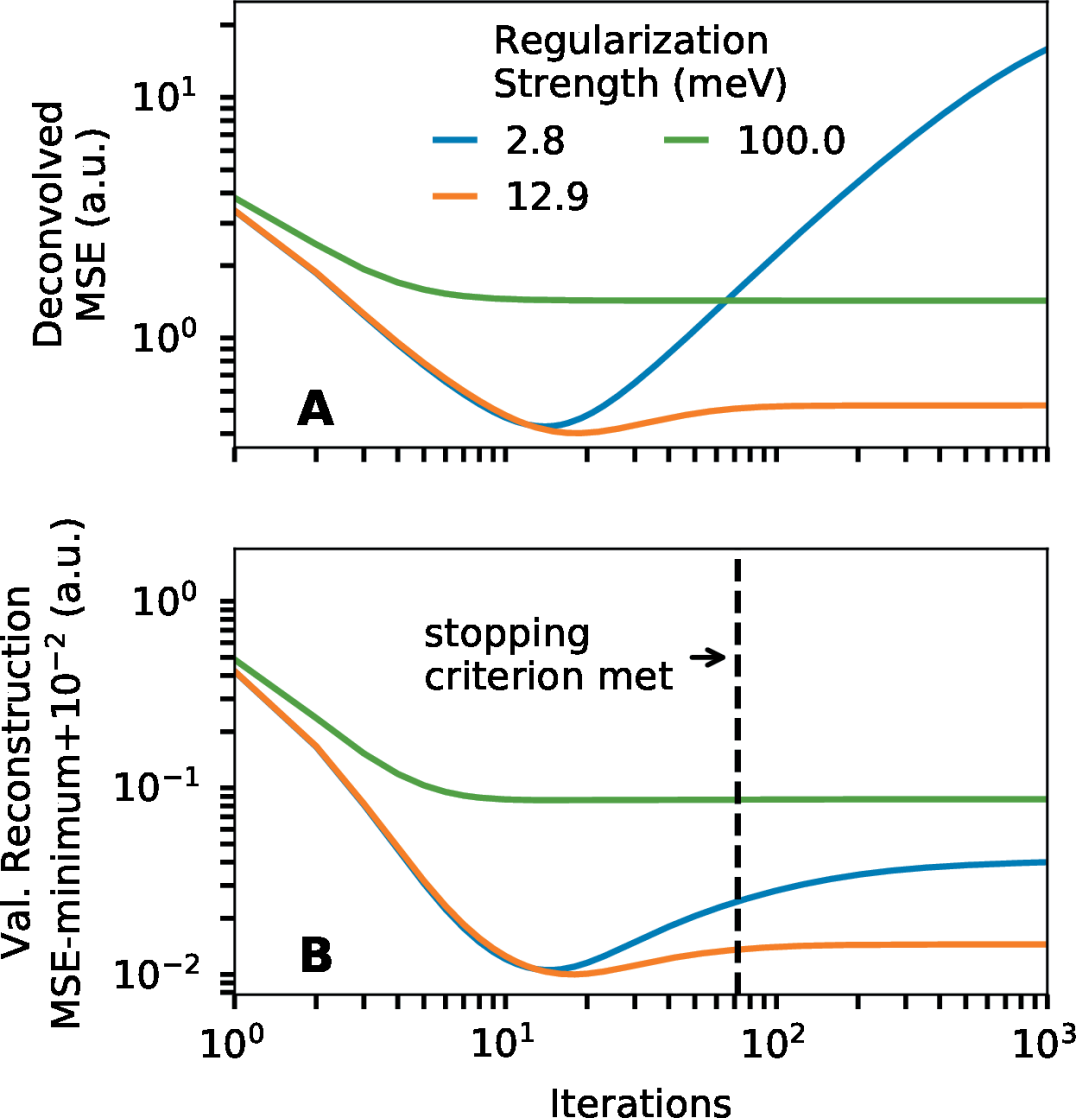
Illustration of the stopping criterion for the same data set as Fig. 3 ▸(A). (A) Dependence of the deconvolved mean squared error (MSE) on iteration number and regularization strength. (B) Dependence of MSE of the reconstruction of validation data on iterations. The stopping criterion is four times the number of iterations required for the validation error to reach a minimum with the smallest tested regularization strength.

Fig. 5 ▸ illustrates the stopping criterion we used for this study. This uses data simulated with the same conditions as Fig. 3 ▸(A). For smaller regularization strengths, the deconvolved and validation reconstruction mean squared errors reach minima with increasing iterations before increasing again. For larger regularization strengths, the deconvolved and validation reconstruction mean squared error decrease to near their asymptotic value quickly without overshooting. We see that, for these data, to fulfill the above conditions, it is necessary to complete iterations at least a few times more than that where the errors for the smallest regularization strength are minimized. In our case we chose to conduct iterations at least equal to four times that where the validation RMSE for the smallest regularization strength reaches a minimum. We note that it can still be beneficial to perform more iterations where possible to better fulfill the above priorities.

**Figure 6:**
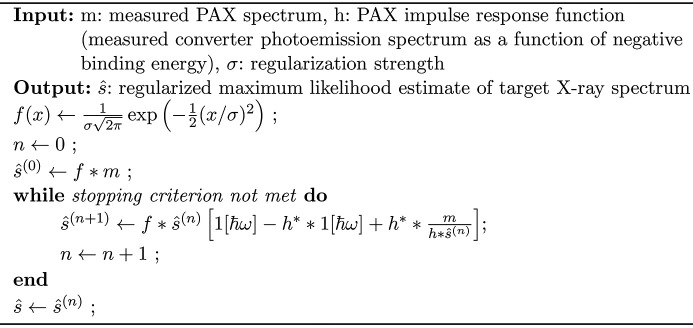
Algorithm 1: regularized deconvolution of PAX data.

We have now finished describing an algorithm for estimating a ground truth X-ray spectrum from PAX data. We summarize this procedure in Algorithms 1 and 2 shown in Figs. 6 ▸ and 7 ▸. The code used to perform the deconvolution analysis described in this report can be found at github.com/dhigley6/PAX2.

## 4. Performance of PAX

Now that we have described an algorithm for estimating RIXS spectra from PAX data, in this section we evaluate the performance of this algorithm on the experimental data of Dakovski *et al.* (2017 ▸) and higher-resolution simulated data. In the simulations, we assume that the bandwidth of X-rays incident on a sample as well as electron analyzer resolution are significantly smaller than the width of the photoemission features used for PAX and thus neglect broadening of spectra from these factors. Real photoemission spectra can have non-uniform backgrounds and satellite photoemission features, but for simplicity these were not included in the simulations we show. Similarly, the model RIXS spectra in the simulations do not include non-uniform backgrounds. We also only included Poisson noise from X-ray counting in the simulation, assuming that this was the dominant noise source. We note, however, that the experimental data have a non-uniform photoemission background and could have other sources of noise, but the RIXS estimation algorithm worked well for it. We simulated PAX measurements using the Ag 3*d* photoemission lines to estimate features with structure on a 100 meV scale as well as a sharp Fermi edge such as seen in Au to estimate finer features.

### 4.1. Experimental data with ∼0.5 eV resolution

**Figure 8:**
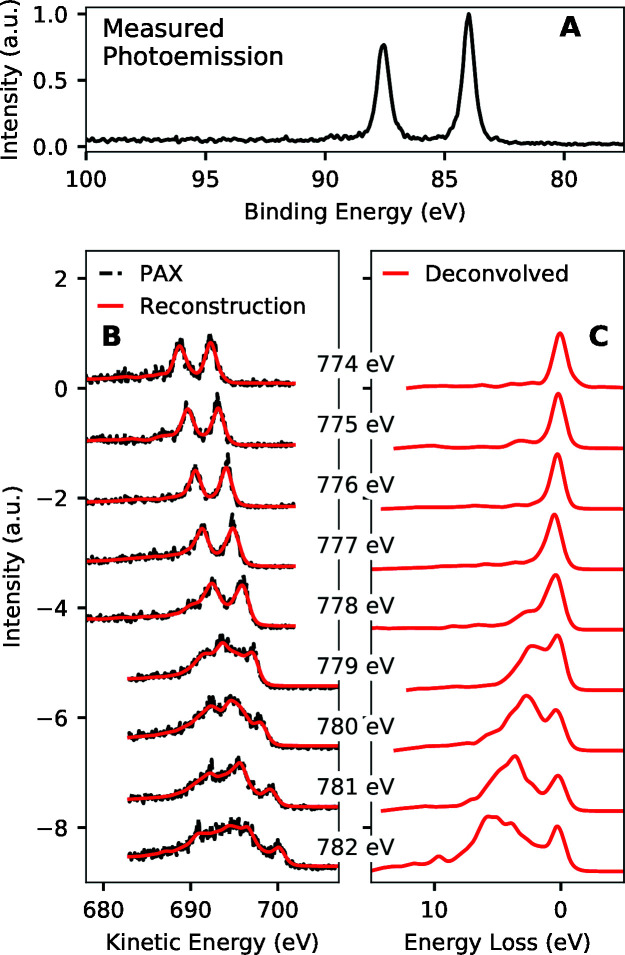
Results of the algorithm described in this report on the ∼0.5 eV resolution experimental data from Dakovski *et al.* (2017 ▸). These data use PAX with the Au 4*f* levels as a photoemission converter to look at RIXS from a CoO sample near the Co *L*_3_-edge. (A) Experimentally measured Au 4*f* photoemission spectrum. (B) Raw PAX data (black) as well as the reconstruction of that data from the deconvolved results (red) as a function of incident X-ray photon energy. (C) RIXS spectra that result from deconvolving the PAX spectra shown in (B).

Fig. 8 ▸ shows the performance of the algorithm we proposed here on the ∼0.5 eV resolution experimental data of Dakovski *et al.* (2017 ▸) recorded at the Linac Coherent Light Source (LCLS). These data used the Au 4*f* lines to convert RIXS photons to electrons and estimated RIXS at the Co *L*_3_-edge of a CoO sample. The regularization strength for the entire data set was chosen by applying the procedure described above to two spectra recorded with 780 eV incident photon energy. The reconstructed PAX spectra of Fig. 8 ▸(B) approximate a smoothed version of the measured PAX spectra. This shows that the deconvolution algorithm accurately estimates the true RIXS spectra within the resolution and electron count limits of the experiment. We note that this performance is achieved even with the non-uniform background of the Au 4*f* photoemission. The estimated RIXS spectra (deconvolved PAX spectra) of Fig. 8 ▸(C) show the expected dependence of RIXS on incident photon energy (van Schooneveld *et al.*, 2012 ▸). The scattering is largely elastic below the absorption resonance (below 778 eV), then transitions to having inelastic loss features that increase in prominence and extend to larger energy losses as the photon energy continues to increase. The data do not reveal the finer structure of the RIXS spectra though, due to the ∼0.5 eV resolution.

### 4.2. Simulated performance estimating features with few hundred mev scale using the Ag 3*d* levels

The Ag 3*d* levels have similar quantum efficiency as the Au 4*f* levels, but narrower intrinsic width. Under such conditions, Dakovski *et al.* (2017 ▸) showed that ∼10^4^ electrons could be collected in 30 minutes at LCLS and estimated that 10^6^ electrons could be collected in a similar amount of time by improving photoemitter surface quality and using a different electron analyzer. The incident X-ray flux would be about an order of magnitude less than Dakovski *et al.* (2017 ▸) for our assumption of both the bandwidth of incident X-rays and electron analyzer resolution being small in comparison with photoemission features to hold, however [Dakovski *et al.* (2017 ▸) estimated 260 meV incident X-ray bandwidth and 295 meV analyzer resolution for the experiments described therein.] At modern synchrotron light sources though, the photon flux per unit bandwidth on a sample can be at least 40 times higher and the count rate correspondingly higher (Strocov *et al.*, 2010 ▸; Dakovski *et al.*, 2017 ▸). Higher number of counts could be obtained by integrating longer (two days of integration for two orders of magnitude more counts than 30 minutes, for example). Thus, here, we simulate the expected performance of PAX using the Ag 3*d* levels for electron counts extending from 10^3^, a count that should be easily achievable in less than 30 minutes even for an experimental setup without improvements from Dakovski *et al.* (2017 ▸), to 10^7^, a count that we expect would currently require integrating for a few hours at a well optimized setup.

**Figure 9:**
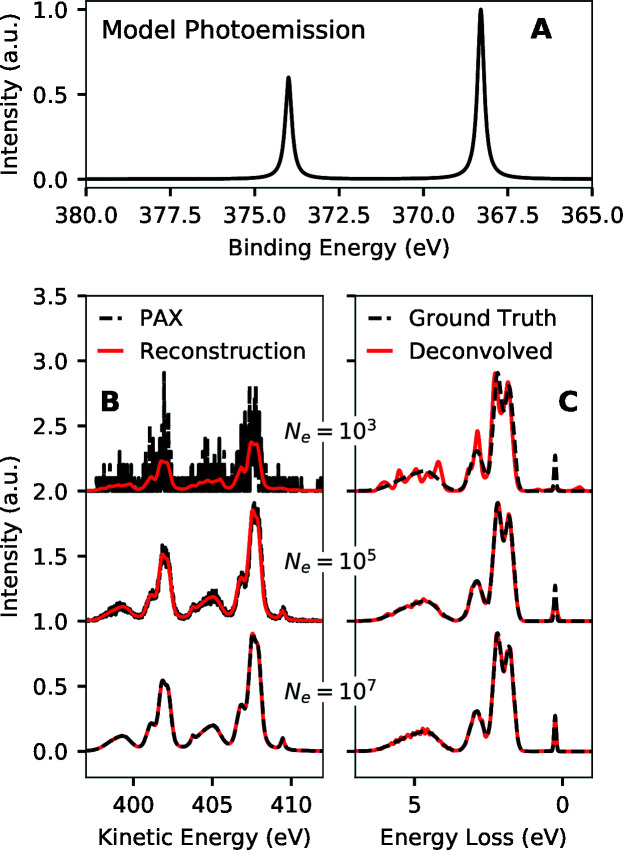
Simulated performance of PAX in estimating a model RIXS spectrum using a model Ag 3*d* photoemission converter. (A) Model Ag 3*d* photoemission spectrum with 233 meV FWHM Lorentzian peaks (Panaccione *et al.*, 2005 ▸). (B) Simulated PAX spectra as well as the reconstruction of that data from the deconvolved results as a function of number of simulated detected electrons in the PAX spectra (*N*_e_). (C) RIXS spectra estimated through deconvolution of the simulated PAX data. The ground truth spectrum is also shown for each case.

Fig. 9 ▸ shows the simulated performance of PAX with the Ag 3*d* lines as a photoemission converter in estimating the model RIXS spectrum shown in Fig. 1 ▸(A). As the number of simulated detected electrons increases, finer details of the X-ray spectrum are accurately estimated. Features with a few hundred meV width are already estimated well with ∼10^5^ simulated detected electrons, and the width of well estimated features decreases further with increasing number of simulated detected electrons.

**Figure 10:**
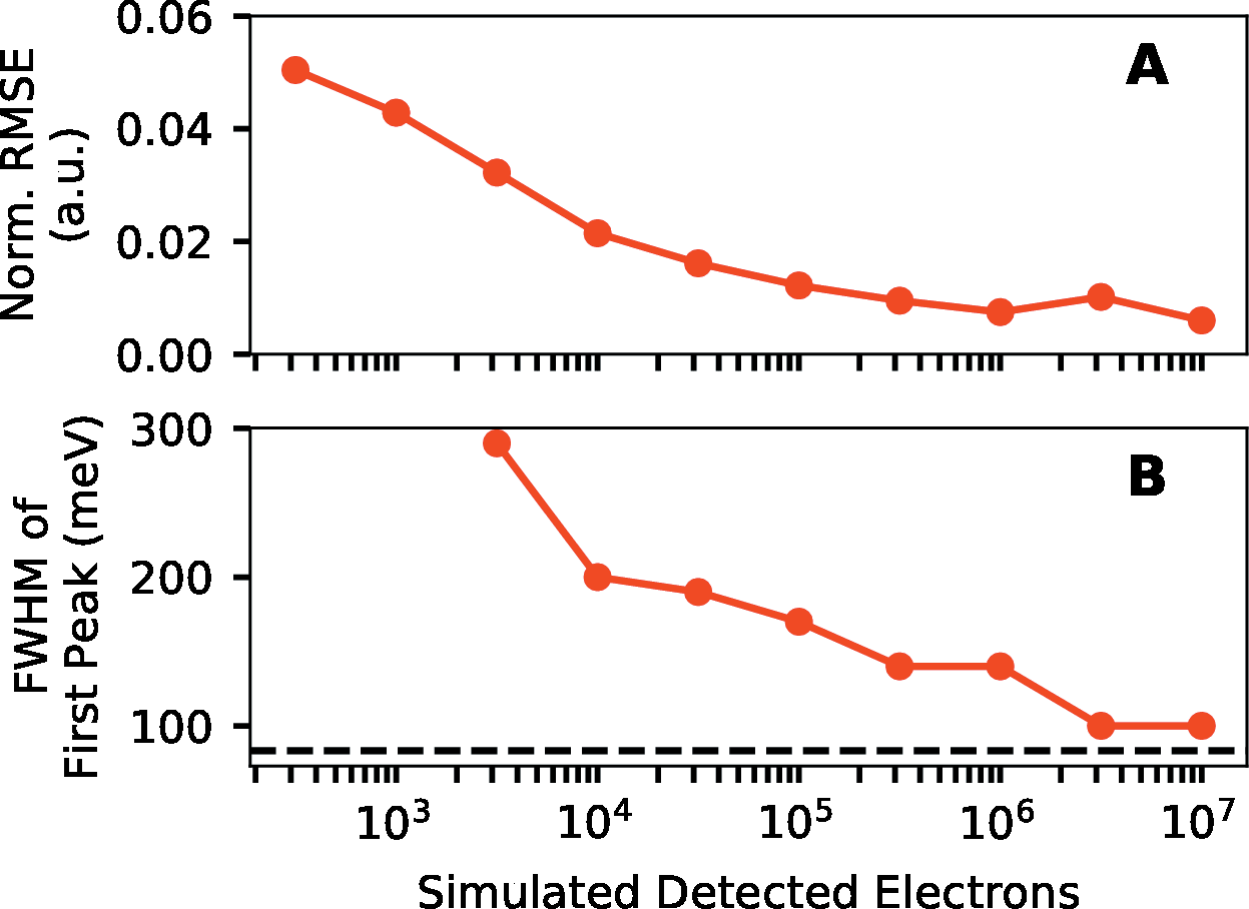
Quantification of the performance of PAX on the simulated data of Fig. 9 ▸. (A) Normalized RMS error (RMSE) of the estimated RIXS spectra versus the number of simulated detected electrons. (B) FWHM of the lowest energy loss peak in the estimated RIXS spectra as a function of the number of simulated detected electrons.

Fig. 10 ▸ quantifies how well the RIXS spectra estimated from simulated PAX data correspond to the ground truth spectrum as a function of the number of simulated detected electrons. The normalized RMSE of the deconvolved spectrum, defined as the RMSE of the deconvolved spectrum divided by the maximum value of the ground truth spectrum, decreases as the number of simulated detected electrons increases, as shown in Fig. 10 ▸(A). Fig. 10 ▸(B) quantifies the ability of the method to accurately estimate fine features through the FWHM of the lowest energy loss peak of the deconvolved spectrum as a function of the number of simulated detected electrons. (Points are not displayed for numbers of detected electrons not sufficiently high to retrieve an estimate of the lowest energy loss peak.) This FWHM decreases as the number of simulated detected electrons increases, and, after 10^6.5^ electrons have been simulated to be detected, the width of the feature in the deconvolved spectrum is within 20% of its true 83 meV width. We note that the integrated intensity of this first loss feature is only 2.4% of the total integrated intensity of the entire model RIXS spectrum. Thus one only needs to have less than 10^4^ photoelectrons simulated to be detected that were emitted from RIXS photons originating from this feature in order to accurately estimate the shape of this feature. We accurately estimated this feature from simulated data despite it being much sharper than the the 233 meV FWHM widths of the Ag 3*d* photoemission peaks of the model impulse response function.

### 4.3. Simulated performance estimating features with tens of meV scale using a sharp Fermi edge

For many interesting cases, particularly for solids (Ament *et al.*, 2011 ▸), it is important to characterize the structure of RIXS spectra on scales finer than 100 meV. For such cases, one can consider using sharper photoemission features to convert X-rays to electrons, although these sharper features usually come at the expense of reduced quantum efficiency. Here, we consider using the Pt valence electrons within 0.5 eV of the Fermi edge, which we model as a sharp edge. The efficiency of photoemission from these states, however, is about three orders of magnitude less than that of the Ag 3*d* or Au 4*f* levels. This, combined with the reduction in incident X-ray bandwidth and electron analyzer resolution (from ∼100 meV to ∼10 meV each) means that the PAX count rate for these electrons can be expected to be about five orders of magnitude lower for the Ag 3*d* or Au 4*f* levels. We simulate 10^5^ simulated detected electrons, which is a count total that would likely take a prohibitively long time to collect at a source like LCLS. At new high-repetition sources like LCLS-II though, the photon throughput is anticipated to increase by three to four orders of magnitude, and such a count total could be realistically reached. We note, however, that this increase in source photon throughput is also expected to improve the count rate of grating-based RIXS studies. Nonetheless, the compactness and relative portability of a PAX setup means it could be used in some cases where a tens of meV resolution grating-based spectrometer could not.

**Figure 11:**
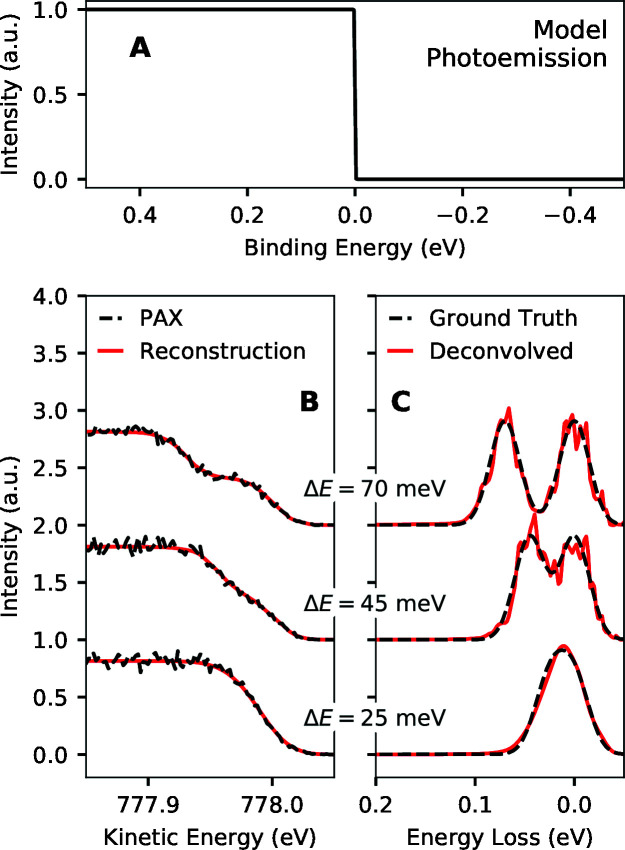
Simulated performance of PAX using a sharp Fermi edge photoemission converter in estimating model doublet RIXS spectra with different peak separations (Δ*E*). For each case, ∼10^5^ electrons were simulated to be detected from photoemission within 0.4 eV of the Fermi level. The doublets consisted of two Gaussian peaks with 100 meV FWHM each and variable peak separation. The shape of the Fermi edge was determined by a Fermi–Dirac distribution at 4 K (boiling point of helium). (A) Model Fermi edge photoemission spectrum for a metal at 4 K (boiling point of helium). (B) Simulated PAX data (black) as well as the reconstruction of that data from the deconvolved results as a function of the peak separation in the doublet (Δ*E*). (C) RIXS spectra estimated through deconvolution of the simulated PAX data and corresponding ground truth spectra.

Fig. 11 ▸ shows the results of simulations assessing the potential of PAX in estimating spectra with structure on scales finer than 100 meV by using a sharp Fermi edge. For this analysis we used an energy separation of 2 meV between points. The photoemission spectrum was modeled with a constant density of states near the Fermi level and a temperature of 4 K (boiling point of He). PAX spectra were simulated for 10^5^ electrons detected from photoemission within 0.4 eV of the Fermi level and variable separation of the X-ray doublet peaks.

As the peak separation decreases from top to bottom in Fig. 11 ▸, our ability to tell that the ground truth spectrum is two peaks rather than a single peak decreases. With the 70 meV peak separation (top) as well as the 45 meV peak separation (middle), it is clear that the deconvolved spectra are not well represented by a single peak. In contrast, for the 25 meV peak separation (bottom), it is no longer clear that the ground truth spectrum consists of more than one Gaussian peak, and a measurement with better statistics would be required to tell this. To obtain an even better idea for experimental data of whether features in an estimated RIXS spectrum are true features and not due to random noise, one could use the bootstrap method (Efron & Tibshirani, 1986 ▸; James *et al.*, 2013 ▸).

## 5. Conclusions and outlook

We have proposed and validated an algorithm for estimating RIXS spectra from PAX data. The algorithm successfully analyzed ∼0.5 eV resolution data originally reported by Dakovski *et al.* (2017 ▸) and performs well on simulated data sets with high resolution. For simuated data using the Ag 3*d* levels as a photoemission converter for PAX, few hundred meV FWHM features could be accurately estimated with 10^5^ simulated detected electrons. Even finer features can be accurately estimated with higher numbers of simulated detected electrons. Details with aspects much smaller than 100 meV could be estimated when a sharp Fermi edge photoemission converter was used in simulations, but at the expense of greatly reduced conversion efficiency of RIXS photons to electrons, and experimentally attaining a useful electron count total for such a measurement will be challenging.

Our proposed PAX deconvolution algorithm is simple and closely linked to the classic Lucy–Richardson algorithm. Recently, however, more sophisticated algorithms have shown promise on achieving more accurate deconvolution results and reducing computational time (Ikoma *et al.*, 2018 ▸; Zhang *et al.*, 2017 ▸). Applying such techniques to PAX could improve on the performance described here. In addition, using uncertainty quantification methods would enable more robust interpretation of X-ray spectra estimated from PAX data. This has been done for similar problems by assessing the sensitivity of deconvolved spectra to artificially added noise (Fister *et al.*, 2007 ▸) as well as more sophisticated methods (Kaipio & Somersalo, 2006 ▸).

Modifications of the experimental setup we propose here could also push the capability of PAX further. The model photoemission converters that we highlighted were chosen based on a survey of literature photoemission data. A systematic investigation of other materials may provide photoemission features better suited for PAX measurements. Finally, the PAX measurement method could be applied to other situations where high signal-to-noise ratio estimates of X-ray spectra are desired without using traditional grating-based technology. An example could be transmissive soft X-ray spectrometers.

## Figures and Tables

**Figure 7:**
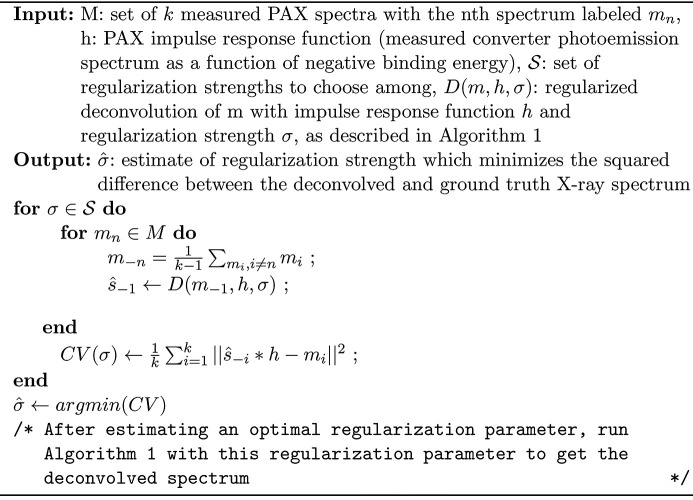
Algorithm 2: estimate optimal regularization strength for regularized deconvolution.

**Table 1:** Description of photoemission from some electronic subshells of solids

Subshell	Description
Ag 3*d*	Two 233 meV FWHM peaks (Panaccione *et al.*, 2005 ▸)
Au 4*f*	Two 335 meV FWHM peaks (Takata *et al.*, 2005 ▸)
Al 2*p*	Two 60 meV FWHM peaks (Borg *et al.*, 2004 ▸)
Pt valence	Sharp Fermi edge
